# Atypical Peripheral Ossifying Fibroma of the Mandible

**DOI:** 10.3390/dj10010009

**Published:** 2022-01-06

**Authors:** Tomislav Katanec, Lea Budak, Davor Brajdić, Dragana Gabrić

**Affiliations:** Department of Oral Surgery, School of Dental Medicine, University of Zagreb, 10000 Zagreb, Croatia; lbudak@sfzg.hr (L.B.); dbrajdic@kbd.hr (D.B.); dgabric@sfzg.hr (D.G.)

**Keywords:** peripheral ossifying fibroma, irritation fibromatosis, gigantocellular lesions

## Abstract

Peripheral ossifying fibroma (POF) is a benign localized lesion originating from gingival and alveolar oral mucosa. Its origin can be cells of periodontal ligament. The lesions usually develop in women in their twenties. POF is a complex clinical and histological diagnosis due to its shared characteristics with many other conditions. In this paper, we presented a case of an atypical peripheral ossifying fibroma (POF) in the left lateral part of the mandible in a 70-year-old male patient who had two semicircular bridges supported on four implants in the upper and lower jaws. A review of CBCT and orthopedic imaging showed no visible intraosseous changes. Histological analysis revealed the diagnosis of POF. The case in question is interesting, as elaborated on in the discussion section of this paper because POF is usually found in female patients aged between 20 and 30 years.

## 1. Introduction

Peripheral ossifying fibroma (POF) is a non-malignant localized lesion originating from gingival and alveolar oral mucosa, clinically manifesting as a painless, slow-growing, hard nodule, usually smaller than 2 cm. It is histologically characterized by a fibrous tissue affected by a variable number of fibroblastic cells. The presence of well-defined islands of metaplastic bone can be extremely helpful when setting a differential diagnosis [1,2]. Various sources name this pathology differently, e.g., peripheral cementifying fibroma, peripheral fibroma with cementogenesis, peripheral fibroma with osteogenesis, peripheral fibroma with calcification, calcified or ossified fibrous epulis and calcified fibroblastic granuloma [1]. The lesions usually develop in women in their twenties and can appear anywhere in the mouth, including the tongue, lips, mouth floor, palate or maxillary and mandibular alveolar crest [3]. A mutation of the SATB2 gene inactivates the gene using different molecular mechanisms, which can lead to the development of this mass. Proliferating cell nuclear antigen-positive cells in POF, indicating the high proliferative activity of the lesion, may influence the treatment modalities. The result is a so-called syndrome related to SATB2 [4], a condition characterized by neurodevelopmental and behavioral disabilities, palatal clefts, teeth and bone anomalies that rarely cause defects in other organ systems [5]. POF is a complex clinical and histological diagnosis because it shares characteristics with many other conditions, such as pyogenic granuloma, peripheral gigantocellular granuloma, irritation fibroma or the non-bone metastasis of some tumors [6]. However, POF is a focal reactive, non-neoplastic tumorous mass of the soft tissue, primarily originating from the interdental papilla. It can be soft and has a smooth surface. The color can vary from a light rosy color to a dark cherry red [7].

## 2. Case Study

**Figure 1:**
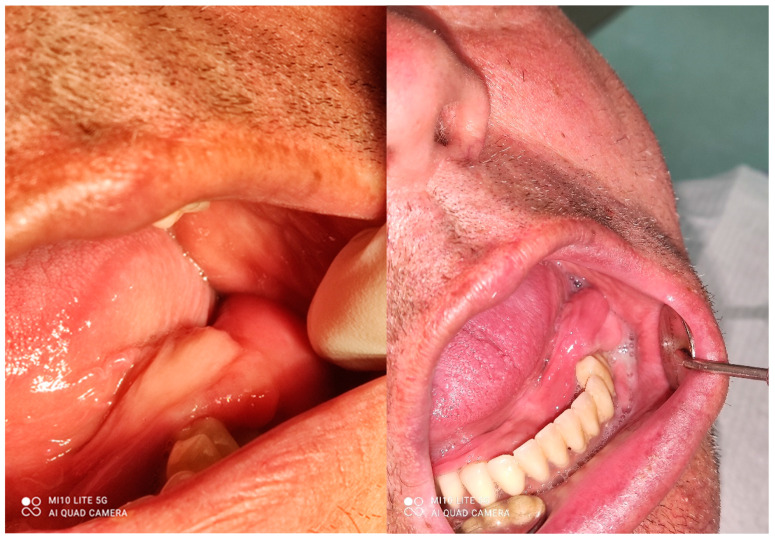
Clinical appearance of the mass during first visit.

**Figure 2:**
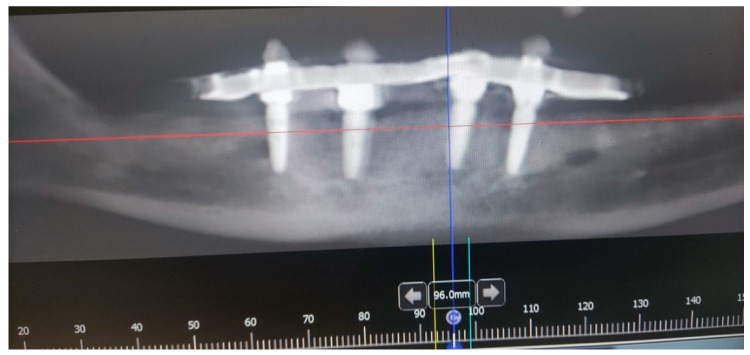
Orthopantomogram of the lower jaw of the patient.

**Figure 3:**
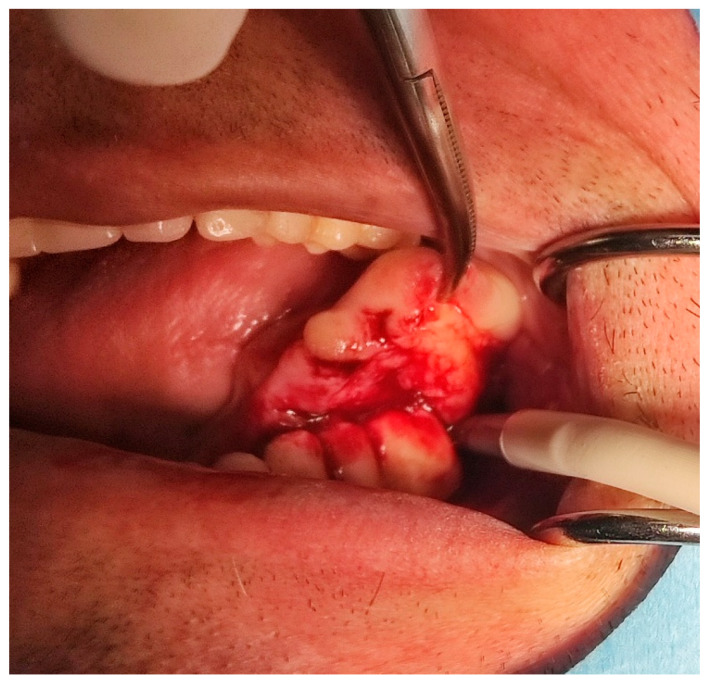
Removal of the mass.

**Figure 4:**
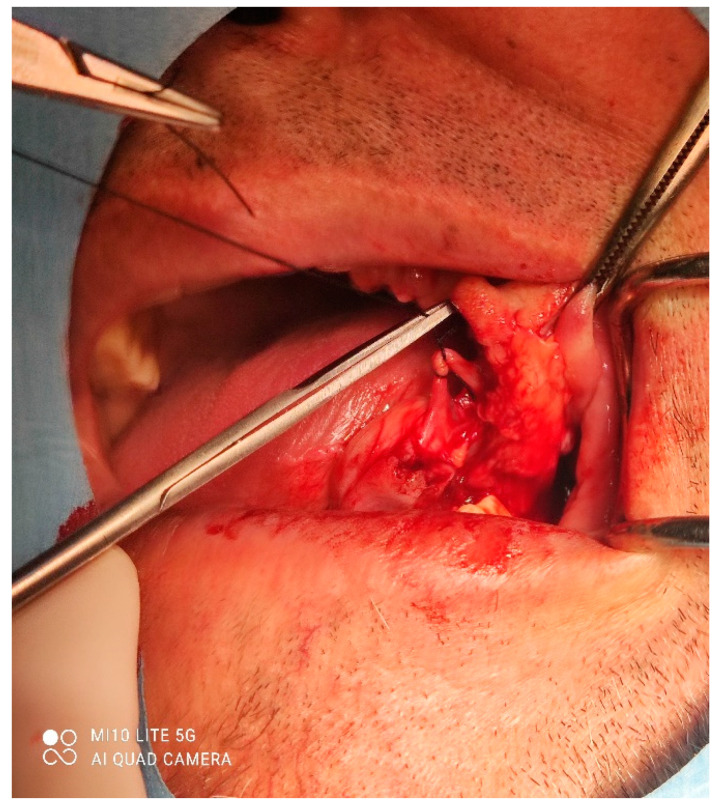
Ligation of the artery.

**Figure 5:**
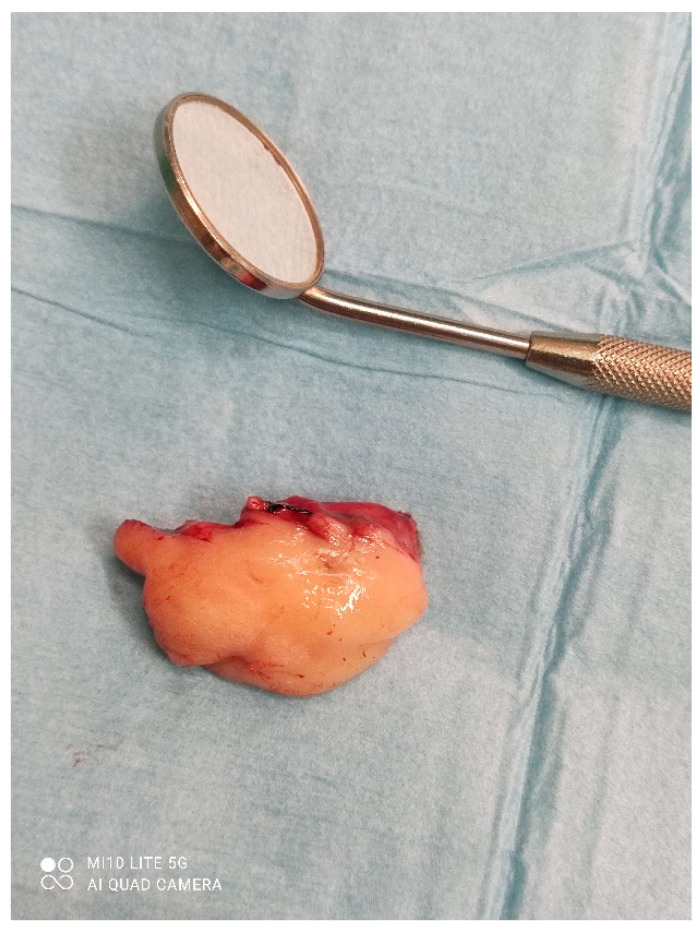
Size of the mass.

**Figure 6:**
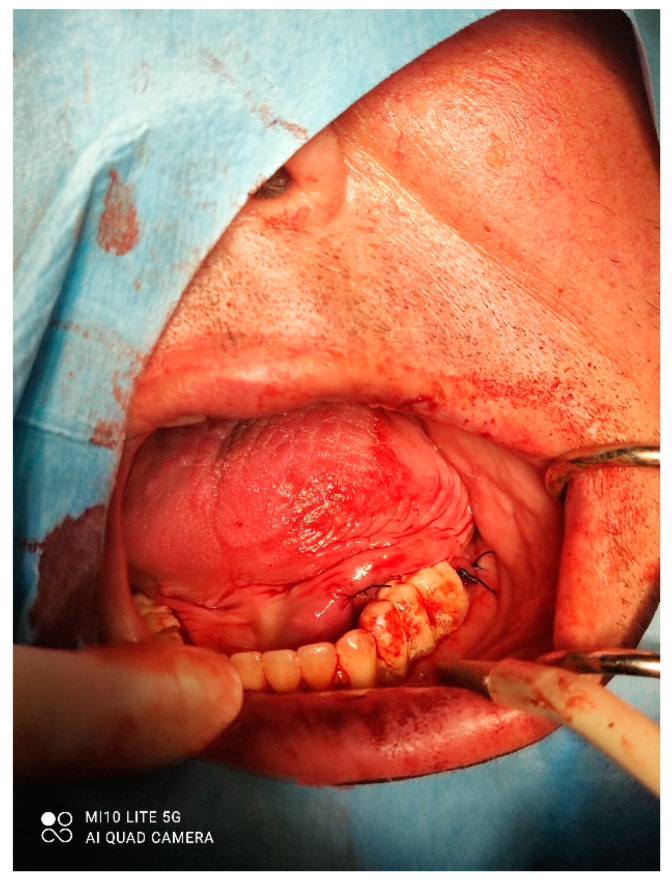
Right after suturing.

**Figure 7:**
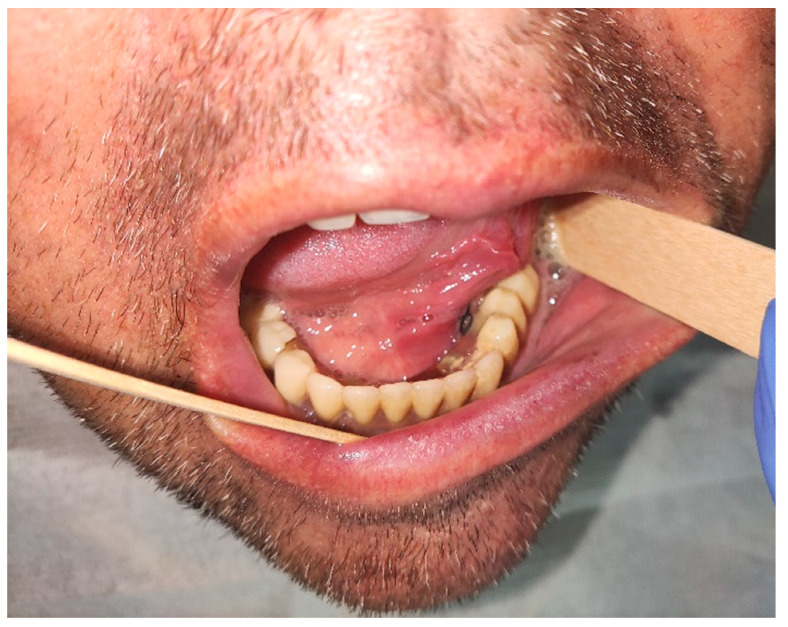
Seven days after operation, medical examination and removal of sutures.

A male 70-year-old patient came to the Department of Oral Surgery, University Hospital Centre Zagreb with a voluminous fibrous mass in the distal region of the left mandible (Figure 1). The patient has two acrylic bridges on four implants. The implants were placed six months prior to admission to the clinic, before the patient noticed the appearance of the mass. The acrylic bridges are 3 months old. The patient states feeling “swelling in the back part of the left mandible three months before coming in for a checkup”. Panoramic radiograph and CBCT did not show any radiolucency, radiopaque areas or signs of periimplantitis around the implants in the bone (Figure 2). The lesion has a smooth surface, with no ulcerations. It was on a broad base connected, with the sublingual anatomical region. The patient has poor oral hygiene, smokes and consumes alcohol: about two to three glasses of wine or beer a day. The palpation of the mass indicated that it was fixed to the alveolar crest of the left mandible on a wide base and was spreading to the left sublingual area. The measured size of the mass was 3.5 × 2 cm. After conducting a clinical examination, the differential diagnosis was possible irritation fibromatosis, peripheral gigantocellular fibroma or peripheral ossifying or non-ossifying fibroma, as well as a malignant mass, and the final diagnosis will be reached after the final PHD analysis. The final decision was an excision in toto (Figure 3). The mass was approached and the layers of submucosa were divided with a scalpel and an electro knife. During operation, it was noticed that the mass has a belonging artery connected to the left sublingual area. The artery was ligated with a resorbing thread 4/0 and the mass underwent complete excision (Figure 4). Parts of the flap were left to heal per secundam, but most of the incision towards the sublingual region was stitched with a non-resorbing silk thread 4/0 (Figure 5 and Figure 6). A full hemostasis was achieved by electrocauterization of the bleeding areas. The excison was performed under local anesthesia. Clinical, medical examination and removal of sutures were performed seven days after surgery (Figure 7).

**Figure 8:**
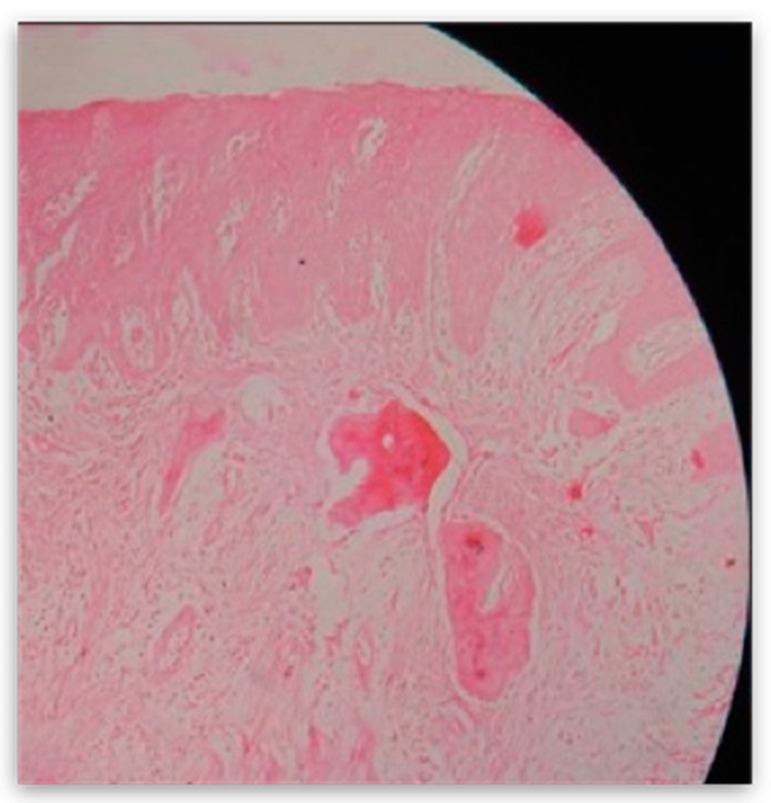
Histological finding of the preparation at magnification 4×.

The mass was then sent for pathohistological (PHD) analysis, which showed that the mass has a reactively changed multilayered epithelium with abundant mononuclear linear inflammatory infiltrate underneath. There were no elements of Lichen. Fragments resembling cemento-osseous lacunae were found inside the thick fiber stroma. A restricted area with clusters of hemosiderin and gigantocellular cells was found in the middle of the sample. Interestingly, a small salivary gland with multiplied intra- and interlobular tissue with widened performing ducts was found in the sample. The outer edges of the sample also contained part of the mucocele wall (Figure 8).

Poorly coordinated intermaxillary relations between the upper and lower acrylic bridge, as well as parafunctional movements, can be considered distinct predisposing factors for the development of this formation.

## 3. Discussion

The appearance of POF is of unknown etiology and can be linked to different susceptibility factors such as bruxism, dental plaque and inadequately set or adjusted prosthetic teeth. Different names for POF are used in the literature, such as fibrous epulis or calcifying fibroblastic granuloma [8]. Peripheral fibroma and fibromatosis are found in relation to CD 34, α-smooth muscle actine (α-SMA), vimentin, Ki-67 (Mib1) and transforming growth factor-α (TGF-α). TGF-α is presumably connected to fibroblast proliferation and fibroblastic activity [9]. POF forms 3.1% of all oral tumors and 9.6% of all gingival lesions [10]. As a reactive benign lesion of the fibrous tissue, POF is not a counterpart of the soft tissue to the central ossifying fibroma, which represents an osteogenic neoplasm. Central ossifying fibroma originates from endosteum or periodontal ligament near the root apex and then spreads in the medullar bone cavity. On the other hand, the peripheral type of the ossifying fibroma, as well as peripheral gigantocellular granuloma (PGCG), collides with the periodontal ligament from which it develops. It can be found solely in soft tissues above the alveolar crest. Clinically, POF resembles a solitary, slow-growing and well-restricted nodular mass with a smooth surface, usually accompanied by normal colored mucosa. It has a wide base and is usually of a solid consistency [11].

Intraoral ossifying fibroma has been described in the literature since the late 1940s. Various names were given to comparable lesions, such as epulis, peripheral fibroma with calcification, peripheral fibroma with osteogenesis, calcified fibroblastic granuloma, peripheral cementifying fibroma, peripheral fibroma with cementogenesis and peripheral cemento-ossifying fibroma [8,12]. Around 60% of similar lesions are found in the upper jaw and more than half of all cases impact the region of incisors and canines, to be exact, the interdental papilla. Usually, children and adolescents aged from 10 to 30 are affected [13,14], but mostly 20-year-olds, with a decreasing incidence after the age of 30 [15]. Only 0.5% of cases occur in older age groups [16]. Due to hormonal influences, women are more likely to be affected by the growth of this lesion [17]. Kfir et al. concluded that the size of POF is usually smaller than 1.5 cm in diameter. A case of a gigantic POF was recorded in the literature, which measured 9 cm in diameter [18]. Therefore, this case is quite interesting because POF was found in a male patient aged 70 who had elevated levels of parathyroid hormone (PTH). 

Ogbureke et al. presented a case of a 44-year-old male who came into the emergency room and complained about swelling in the back segment of the right mandible that had been going on for three months. His family history included diabetes mellitus type 2, cardiovascular diseases and hypertension. He had two implants in the right distal quadrant near the lesion three months before the appearance of the mass. It was concluded that it was quite hard to clinically differentiate peripheral gigantocellular lesion and peripheral ossifying fibroma. The difference can be established histologically. Gigantocellular cells are present in both POF and peripheral gigantocellular granuloma, whereas cemento-ossifying lacunae is only present in POF [19].

Gulati et al. reported a case of a female, 56-year-old patient with a mass of hard consistency upon palpation. The dimensions were 3.5 cm × 4 cm × 3 cm. The mass was located near teeth 13–23 and the teeth were completely periodontally compromised (stage III). A mass had a wide base and was erythematous and painless. The panoramic radiograph showed a clean radiographic status. 940-nm diode laser (Biolase^®^, Foothill Ranch, CA, USA) was used to remove the mass. Pathohistological analysis showed fibro-cellular stroma with scattered islands of osteoid tissue varying in size (both mature lamellar bone and immature bone). Calcification of the bone tissue showed peripheral osteoblastic margins in some places. Some proliferations of the endothelial and inflammatory cells were present in the tissue. Overall, the pathohistological characteristics indicated POF [20]. 

Prasad et al. believe that POF can develop as a pyogenic granuloma in the beginning, which later goes through maturation of the fibers and calcification [21]. 

Satish et al. claim that POF is a consequence of inflammatory hyperplasia of the periodontal cells or periost. Metaplasia of the fibrous tissue leads to dystrophic calcification and the formation of the bone [22].

Rallan et al. show the case of a twelve-year-old boy who had taken notice of the swelling that started a month before his visit to the department of pediatric dentistry and observed that it increased in size. The patient’s medical history did not point to any significant health indications. An oval-shaped gingival mass was discovered in the palatal region of maxillary incisors upon completion of the intraoral examination. The mass was impeding his bite and distressed the patient. The swelling was well-circumscribed, sessile, erythematous, firm on palpation and measured approximately 2 × 2 cm in dimensions. The lesion was asymptomatic with no clinically visible ulcerations.

They concluded that the mass is a reactive lesion of the connective tissue. Predisposition to such lesions is most common in the anterior maxilla of young women. The standard treatment protocol involves excisional biopsy, followed by histopathological evaluation. As the lesion has a tendency for recurrence, follow-up is of the utmost importance in most cases [23].

Nadimpalli and Kadakampally describe the case of a 23-year-old female patient with recurrent peripheral ossifying fibroma located in the right lower premolar region. Clinical, radiographic and histologic features of POF, including differential diagnosis, treatment and follow-up, are explained in the report. Due to the possible recurrence of POF, early diagnosis, followed by surgical excision and the curettage of surrounding tissue, are essential measures of recurrence prevention. They noted the importance of early diagnosis and conservative management of such lesions, as they can become more destructive over time if left untreated. Regular follow-up is essential after excision due to the high growth potential of the lesion (8–20% recurrence rate) [24].

Interestingly, Sudhakar et al. show the case of a 55-year-old woman with a mass in the upper jaw in the second left incisor. An intra-oral periapical radiograph (IOPAR) and orthopantomography did not reveal any pathological changes except for generalized horizontal bone loss. Similar to the pathohistological finding in the patient shown in our case, the underlying connective tissue was highly cellular, with plump fibroblasts intermingled in a delicate fibrillar stroma associated with areas of woven tra-becullar bone and osteoids.

Furthermore, due to the gender of the patient and the second decade predilection, the role of hormones has also been questioned as the predisposing factor of POF [25]. 

It is important to point out that, in our case the patient, is a man in his seventies, with the appearance of POF in the area of the left lateral part of the mandible. In the literature that we have already mentioned, the occurrence of POF as well as other ossifying or cemento-ossifying fibromas is more common in females aged from 20 to 50 years. Predilection sites of neoplasms are most common in the front of the maxilla or in the area of the hard palate in the distal parts of the maxilla.

Agarwal et al. Present a 68-year-old female patient troubled by a soft tissue growth on her left palate. The patient noticed a small nodule that grew to the present size in 4 months. The patient had no significant medical and personal history. Clinical intraoral examination discovered a shiny, rounded and elongated pink enlargement on the left side posterior of the palate in projection of the cementoenamel junction of 26. It extended from 23 to 27 anteroposteriorly and was 1 cm lateral to palatal midline to the occlusal surface of the left maxillary molars, buccolingually. Histological examination revealed connective tissue stroma with a rich fibroblastic nature and overlying epithelium comprising bony trabeculae with osteoblastic rimming of mature bone with a mostly lamellar structure, confirming the lesion as POF. On regular follow-up, the lesion healed without any complications [26].

Katanec et al. published a similar case of symmetrical fibrous hyperplasia of the palate. 

In that case, a 47-year-old patient developed a bilateral mass in the hard palate, spreading to the junction of the hard and soft palate. A fibromatous mass appeared 3 years before the visit to the clinic. One year before the clinical examination, the nodule grew to form a voluminous fibromatous mass larger than 5 cm in diameter on both sides. The mass affected the area of the upper canines on both sides to the border with the soft palate. The formation was hard to palpation and connected at a wide base to the palatal artery. No signs of acute inflammation were present. Excision of the formation without interference with healthy tissue, followed by the removal of the affected periosteum and periodontal ligament, was the most suitable treatment. It is of the utmost importance to limit the irritating factors and possible trauma to the tissue [27].

Dutra et al. concluded that the incidence of hyperplastic lesions in all oral pathogenesis is high. It is more common in the female population on the gingiva in the anterior part of alveolar ridge. Inflammatory fibrous hyperplasia is the most common lesion (72.09%), followed by oral pyogenic granuloma (11.79%), giant cell fibroma (7.30%) and peripheral ossifying fibroma (5.24%) [28].

Borghesi et al. present the appearance of four benign formations in the same hemimandible, diagnosed by CBCT in a 50-year-old female patient. That was the first case in the literature showing peripheral osteoma (PO), compound odontoma (CO), focal cemento-osseous dysplasia (FocCOD), and cemento-ossifying fibroma (COF) together in the same patient mandible. This research, as well as our case, is of great importance for the case of differential diagnosis of neoplasms in the oral cavity and bones [29].

In a retrospective survey study and literature review, Sangle et al. suggest that traumatic fibromas are the clinically most common lesions in oral cavity. As we have already stated, Sangle et al. concluded that irritating factors are important predisposing elements for the occurrence of fibromatous formations [30].

## 4. Conclusions

We presented a case report of a 70-year-old male patient who had histologically confirmed POF in left mandible lingually. As elaborated in the discussion of this paper, this case is interesting because POF is more often found in female patients aged from 20 to 30 years. This presented case report is interesting as the patient is an adipose man in his seventies, who has two Toronto bridges placed on four implants in his upper and lower jaw. The occlusion of the bite surfaces of the bridges was not adequately aligned, which led to irritation and was a possible predisposing factor for the development of irritative fibromatosis, i.e., POF in this case.

Overall blood count showed elevated parathyroid hormone levels. This fact may also be related to the formation of POF. After surgical excision of the formation, the operated area of the patient healed properly. There are no signs of recurrence at present, and the occlusion of the existing bridges is properly coordinated.

## Data Availability

For data information contact corresponding author.
